# Implementing Logic Gates for Safer Immunotherapy of Cancer

**DOI:** 10.3389/fimmu.2021.780399

**Published:** 2021-11-04

**Authors:** Mohammed Azharuddin Savanur, Hadas Weinstein-Marom, Gideon Gross

**Affiliations:** ^1^ Laboratory of Immunology, MIGAL - Galilee Research Institute, Kiryat Shmona, Israel; ^2^ Department of Biotechnology, Tel-Hai College, Upper Galilee, Israel

**Keywords:** Boolean logic gates, adoptive cell therapy, chimeric antigen receptors, on-target off-tumor toxicity, synthetic biology

## Abstract

Targeting solid tumors with absolute precision is a long-standing challenge in cancer immunotherapy. The identification of antigens, which are expressed by a large fraction of tumors of a given type and, preferably, across various types, but not by normal cells, holds the key to developing safe, off-the-shelf immunotherapies. Although the quest for widely shared, strictly tumor-specific antigens has been the focus of tremendous effort, only few such candidates have been implicated. Almost all antigens that are currently explored as targets for chimeric antigen receptor (CAR) or T cell receptor (TCR)-T cell therapy are also expressed by healthy cells and the risk of on-target off-tumor toxicity has remained a major concern. Recent studies suggest that this risk could be obviated by targeting instead combinations of two or more antigens, which are co-expressed by tumor but not normal cells and, as such, are tumor-specific. Moreover, the expression of a shared tumor antigen along with the lack of a second antigen that is expressed by normal tissues can also be exploited for precise recognition. Additional cues, antigenic or non-antigenic ones, which characterize the tumor microenvironment, could be harnessed to further increase precision. This review focuses on attempts to define the targetable signatures of tumors and assesses different strategies employing advanced synthetic biology for translating such information into safer modes of immunotherapy, implementing the principles of Boolean logic gates.

## Introduction

In adoptive cell therapy (ACT) of cancer, great effort is made to develop off-the-shelf genes, designed for redirecting autologous T or NK cells to selectively eradicate tumor cells while avoiding on-target off-tumor attack (1–3). Ready-to-use, donor-derived T/NK cells genetically reprogrammed to recognize a given type of cancer will vastly accelerate treatment and assure quality and quantity of the cell product (4). Chimeric antigen receptors [CARs, originally developed by G. G. and colleagues (5, 6)] redirect T, and other immune killer cells to recognize antigens in an MHC-independent manner and are thus ideally suited for this purpose. However, the vast majority of CAR antigens expressed on solid tumors or cellular components of the tumor microenvironment (TME) that are presently investigated are expressed by vital nontumor tissues (7–11) and their targeting poses a severe safety concern. This critical downside has prompted the search for combinations of antigens (present or absent) as well as non-antigenic cues, which could define new targetable tumor-specific signatures. To achieve this goal two major challenges are concomitantly addressed: 1) Identification of combinations, which are shared by the vast majority, if not all tumor cells in a large fraction of patients and can unequivocally discriminate between tumor and nontumor tissues. 2) The development of molecular devices, which can sense and integrate these complex inputs to produce the desired biological output, implementing the basic principles of Boolean logic gates. Two types of logic gates that are particularly relevant to the safety challenge are the logic AND gate, which would only produce an output in the presence of all its designated inputs and the NOT gate, which would require the presence of at least one input and the absence of another, to act. Here we examine different classes of such cues as potential components of tumor-specific signatures and review various strategies used to exploit the AND and NOT gates for their immunotargeting. Other powerful genetic approaches implementing the OR logic gate (12–15), TRUCKs (16–18) or switch receptors (19–22) address additional critical challenges in ACT and are not dealt with in this review.

## Potential Logic Gate Inputs

### Antigenic Cues

While distinct input and output modules of logic gated ACT are defined by their biological thresholds, each intact circuit should eventually comply with clinical tolerability. This aspect is particularly important while weighing the actual expression of a given antigen by tumor vs. normal cells, as the outcome of antigen binding critically depends not only on its expression level, but also on the overall avidity of interaction, the fidelity of the signaling cascade and the response mechanism. With this in mind, attempts are made to identify combinations of surface antigens that are expressed by all cells in a given tumor and by most tumors of a given type and, desirably, by different tumor types, but not by any vital tissue. As the number of input signals grows, precision is expected to increase, but at the expense of the fraction of tumors complying with all inputs and of applicability, as additional modules would inevitably require increasingly improved gene transfer vectors.

Acute myeloid leukemia (AML) is a heterogeneous class of diseases characterized by particularly high genetic instability that gives rise to multiple immunophenotypes in a single patient, rendering AML notoriously refractory to conventional immunotherapies (23). In attempt to circumvent this heterogeneity, Perna et al. (24) performed meticulous proteomics and transcriptomics analysis of large surfaceome datasets from malignant and normal tissues, aiming at identifying antigen pairs which could serve as targets for combinatorial CAR therapy. While a number of antigens have been previously reported as potential AML CAR targets, none met the criteria defined in this study for an optimal single target. Following a stringent refining process, four antigen pairs: ADGRE2+CD33, CCR1+CLEC12A, CD70+CD33, and LILRB2+CLEC12A, yielded nearly optimal results, for all of which the dual targeting score significantly exceeded that of either antigen alone.

More recently, Dannenfelser et al. carried out a comprehensive in-silico screen of >2.5 million dual and 60 million triple antigen combinations across 33 tumor types and 34 normal tissues, assessing their ability to discriminate tumors from normal tissues *via* either AND or NOT gates (25). For prioritizing candidate antigen combinations the authors created a numerical ruler, integrating precision and recall (fraction of targetable tumor samples), with which they compared single antigens to double and triple gates. For every cancer type assessed they identified at least 25 high-score antigen pairs, which substantially outperform single ‘clinical’ antigens currently explored in CAR therapy of the same cancer. Furthermore, for several tumor types, triple antigen combinations show near ideal precision, yet, with an anticipated decline in recall. Although this study is based on RNA-seq databases rather than actual surface protein expression, it underscores the discriminatory power of combinatorial antigen recognition, paving the way for clinical evaluation of numerous CAR therapies targeting new antigen combinations in virtually all cancer types.

Another class of candidate antigens for combinatorial recognition are expressed by tumor-supporting cells at the TME, including cancer-associated fibroblasts, tumor-associated macrophages, myeloid-derived suppressor cells, regulatory T cells and additional immune cells (9–11), as well as tumor endothelial cells (TECs) (26–29). Antigens that are preferentially expressed by these cells (such as VEGFRs, PSMA, ALCAM, CD13, CLEC14A, RGS5, TEMs, fibronectin EIIIB splice variant, Endothelin B receptor, α_v_β_3_ integrin and others) are particularly attractive, as, unlike tumor antigens, they are not subjected to genetic and epigenetic instability. Among these, TECs draw special attention, as they are highly accessible to the therapeutic cells and are less affected by immune-suppressive conditions dominating the TME. While none of these antigens is utterly specific, collectively they provide an additional layer of potential targets for gated therapy.

Loss of heterozygosity (LOH) characterizes the majority of human cancers. It is manifested in multiple losses of full chromosomes, entire chromosomal arms or sub-chromosomal regions and is often associated with the loss of a normal copy of a tumor suppressor gene (30–33). Many LOH events already occur prior to malignant transformation creating a loss signature that is shared by premalignant cells and all descendant tumor cells in a given patient (34). An inevitable outcome of LOH is the concomitant loss of all other genes residing on the deleted chromosomal material, and these naturally include heterozygous alleles of protein-coding genes. This early LOH-driven antigenic landscape is consequently irreversible and is not affected by tumor heterogeneity. LOH-based discrimination of tumor from normal cells can be achieved by NOT gates based on either CARs that are directed at an allelic variant encoding an extracellular epitope, which can be distinguished from the homologous one or TCRs, recognizing a linear peptide that is presented on normal but not tumor cells by one of the patient’s HLA-I products. A special class of LOH events involve the HLA gene locus on chromosome 6q21 and are apparently exploited by tumors as an escape mechanism from CD8 T cells (35–37). It now emerges that this mechanism also allows tumor cells to avoid recognition by adoptively transferred T cells, as reported, for example, in TIL therapy of a patient with metastatic colorectal cancer, targeting a peptide harboring the KRAS G12D mutation presented by HLA-C*08:02 (38). LOH is source for a universe of antigenic combinations that can be targeted by NOT modules and pioneering attempts to explore this new route to cancer immunotherapy have recently been published (39, 40).

### Non-Antigenic Cues at the TME

Hypoxia is a prominent feature of the TME, resulting from rapidly dividing cancer cells and aberrant vascularization (41). Tumor cell adaptation to hypoxic conditions is governed by hypoxia-inducible factors (HIFs) *via* the hypoxia pathway (42). Under normoxic conditions, prolyl-hydroxylases (PHDs) hydroxylates conserved prolines of HIF-1α, leading to ubiquitination and proteasomal degradation. However, during hypoxic conditions, the PHDs are inactive, allowing HIF-1α accumulation, translocation to the nucleus, dimerization with HIF-1β and formation of a transcriptional complex. The HIF complex binds to the promoter region of hypoxia-responsive elements (HRE) and triggers transcription, which regulates several biological functions of tumor cells (43). These two hypoxia-induced mechanisms, acting independently at the transcription and post-translational levels, can be exploited for confining CAR activity to hypoxic tissues.

Pro-inflammatory cytokines secreted by a variety of cells at the TME induce cellular responses that are associated with tumor initiation, progression, tissue invasion, metastasis and evasion from an immune response. In parallel, potent immunosuppressive mediators that are overproduced by tumor-supportive immune cells act in many solid tumors to counteract the antitumor response. Consequently, the TME is often characterized by elevated levels of cytokines, including TNF-𝛼, IFN-γ, IL-6, IL-8, IL-17, IL-21, TGF-𝛽 and IL-10 (44–46), which can serve as TME biomarkers and guide AND gated therapies exploiting cytokine-responsive receptors or transcriptional control elements.

Continuous interactions between tumor and nontumor cells at the TME result in the upregulation of a battery of pericellular proteases, which play a critical role in tumor angiogenesis, invasion and metastasis (47–49). These include various matrix metalloproteinases, cathepsins, elastase, granzyme B, tissue and urokinase plasminogen activators and others, creating a protease-rich niche that is distinguishable from most other normal tissues. This unique characteristic of the TME paves the way for the design of antitumor antibodies or CARs whose recognition moieties are masked by a protease-cleavable peptide, which is liberated at the tumor site, creating a bona-fide logic AND gate.

Tumor-specific as well as tissue- or state-specific promoters can be harnessed to confine the expression of therapeutic genes of interest (GOIs) to designated target cells in-vivo and, as such, offer another class of cues for gated cancer therapy. However, as with tumor antigens, no single promoter appears to be solely restricted to a given type of tumor. Nonetheless, dividing the specificity task between two promoters, both simultaneously active only in the target cells, could confer the desired precision of action (50–52). In addition, synthetic promoters comprising distinct transcription factor (TF)-binding elements can assure that transcription of a therapeutic gene takes place only in cells expressing all corresponding TFs (53, 54).

## Selected Strategies

### AND Logic Gates

#### Combinatorial Antigen Recognition

**Figure 1:**
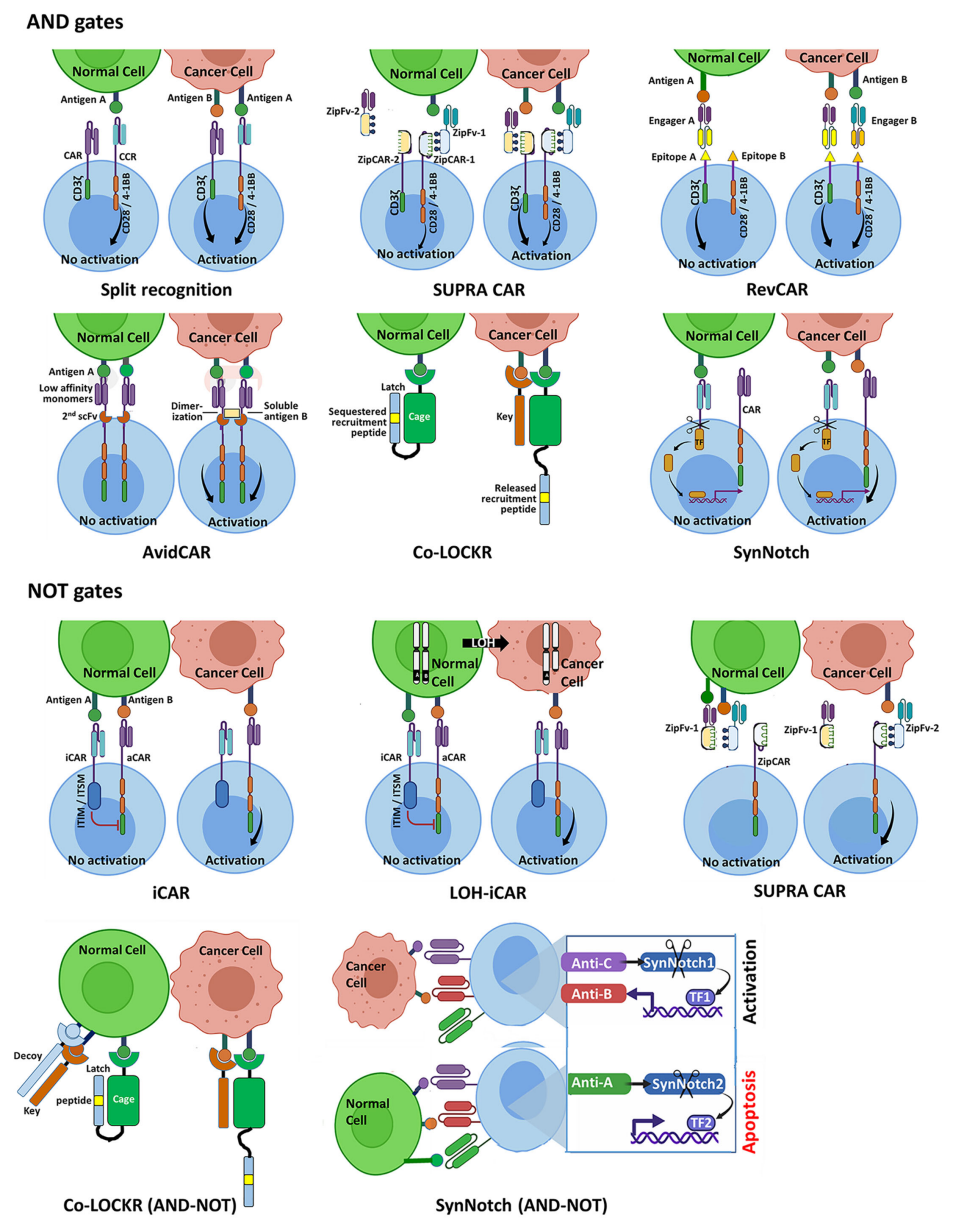
Schematic representation of selected Boolean logic AND and NOT gate strategies for integrating two or three antigenic cues, designed to enhance precision of CAR-T cell therapy of cancer. **AND gates**. The split recognition strategy involves one receptor with an activation domain and a second receptor with a co-stimulatory domain, each specific to a different antigen. Full T cell activation is only achieved upon simultaneous engagement of the two receptors with their respective antigens. Each SUPRA CAR system utilizes a universal CAR-like receptor (zipCAR) carrying a leucine zipper, which can bind a corresponding leucine zipper incorporated onto a soluble antigen-specific zipFv protein, enabling multiplexed control of T cell responses. Shown here are zipCAR-1, equipped with a leucine zipper specific to that of zipFv-1, which recognizes antigen A that is shared by the tumor and normal cell, and zipCAR-2, specific to zipFv-2 and to antigen B that is expressed by the tumor cell but not by the same normal cell. RevCARs are economical CAR constructs, harboring peptide epitopes instead of scFvs. The RevCAR platform utilizes bi-specific engagers, each comprising two scFvs: one specific to a distinct peptide epitope and the other recognizing a tumor-associated antigen. Shown here are a T cell co-expressing two different RevCARs, each harboring a different peptide epitope and either T cell activation or costimulatory domain, and two engagers, each directed at a given peptide epitope and a tumor antigen. The T cell can only be activated when the two engagers are simultaneously bound to their tumor antigens. AvidCAR is an avidity-controlled CAR platform, designed to homodimerize upon dual antigen recognition conferring sufficient avidity for T cell activation. The Co-LOCKR gate utilizes a universal CAR specific to a recruitment peptide, which is placed in a ‘Latch’ sequence. The latch itself is linked to a soluble ‘Cage’ protein that is directed at first tumor antigen. The recruitment peptide is normally sequestered by the Cage and is only exposed to engagement by the CAR when a competing ‘Key’ protein binds a second antigen present on the same tumor cell. The synNotch AND gate comprises a constitutively expressed module which releases a synthetic TF only upon binding of antigen A. Upon translocation to the T cell nucleus, this TF drives the expression of a conventional CAR specific to antigen B. **NOT gates**. The iCAR and the LOH-iCAR typically utilize an inhibitory element derived from a T/NK cell inhibitory receptor. Binding of the iCAR to the inhibitory antigen would prevent T cell activation that could otherwise take place following engagement of the aCAR with the activating antigen. The SUPRA CAR NOT module operates through competition: when both zipFv-1 and zipFv-2 proteins bind their respective antigens A and B on normal cells, their complementary leucine zippers are tightly engaged, precluding activation of the zipCAR. T cell activation can only take place upon encounter with tumor cells, in the absence of competition by non-bound zipFv-1. The Co-LOCKR NOT circuit implements a combination of AND and NOT gates and functions through competition, employing a third protein (in addition to the Cage and Key proteins) that binds specifically to a third antigen present on normal but not tumor cells. This protein possesses a Decoy element, which binds Key with high affinity. When all three proteins are engaged with their target epitopes on the surface of a normal cell, Key is prevented from binding to Cage so that release of the recruitment peptide does not take place. The synNotch circuit shown here utilizes a suicide module, which is only activated upon encounter with an inhibitory antigen expressed by the normal cell to be protected.

The ‘split’ recognition AND gate (**Figure 1**) comprises an affinity-reduced CAR specific to one antigen that harbors an activation domain (typically CD3ζ) only, co-expressed with a chimeric costimulatory receptor (CCR) that is specific to another antigen, which only possesses a costimulatory element (often derived from CD28 and/or 4-1BB), but no activation domain. If properly calibrated, split antigen recognition would allow full T cell activation only in the presence of both antigens, while normal cells expressing only one would be protected. This principle has been demonstrated in several preclinical studies [e.g (55–58)].

SUPRA CARs (**Figure 1**) logically respond to multiple antigens without the need of re-engineering T cells specifically for each target antigen (59). The universal zipCAR module possesses a conventional CAR signaling moiety connected to an extracellular leucine zipper. The zipFv antigen-recognition module harbors a scFv fused to a second leucine zipper, which is capable of interacting with the zipCAR. The engineering of multiple zippers with varying binding properties enables multiplexed control over T cell responses, including a split-CAR configuration.

The RevCAR platform (**Figure 1**) (60) consists of compact CARs incorporating peptide tags instead of full-length scFv, and bispecific molecules coupling an anti-tag scFv to an antitumor scFv. Using two split anti-tag CARs and the corresponding bispecific engagers the system operates as an AND gate.

Reduced affinity CARs are also the central components of the AvidCAR AND gate strategy (**Figure 1**) (61). It exploits monomeric, reduced affinity CARs specific to one antigen, designed to homodimerize by the second antigen through a second scFv incorporated into the same ectodomain, thereby gaining sufficient avidity for T cell activation.

The ‘Co-LOCKR’ gate (**Figure 1**) (62), is based on competition, rather than complementation. This system employs a universal CAR recognizing a unique ‘recruitment peptide’ which resides on a ‘Latch’ domain engrafted onto a protein termed the ‘Cage’ that is capable of binding antigen A. In both the unbound and the bound states of the Cage the recruitment peptide is sequestered by the Cage-bound Latch and cannot bind its receptor. The second soluble protein component of this system is the ‘Key’. It recognizes antigen B and contains a unique domain, which can compete out the folded sequestering domain of the Cage and liberate the recruitment peptide that is now accessible to the CAR. This productive interaction can only take place if antigens A and B are present in close proximity on the same target cell and does not occur in solution.

#### AND Gates Utilizing Synthetic Promoters and Non-Antigenic Cues

The synthetic Notch (synNotch)-based AND gate, (**Figure 1** (63–66), and see (67) for review), harnesses regulated intramembrane proteolysis (68), a unique signaling pathway that utilizes the dual cleavage of a group of cell surface receptors, including Notch, by membrane-associated proteases following ligand binding. This process releases the intracellular domain (ICD), which can then translocate to the cell nucleus and function as a TF. The basic synNotch module encodes an N-terminal scFv targeting antigen A, fused to a minimal Notch sequence including the cleavable transmembrane domain and a microbial-derived ICD, which can operate as a TF that controls gene expression from a synthetic promoter. The second module encodes a conventional CAR targeting antigen B and is placed under the control of this synthetic promoter, so that the entire circuit only operates in the presence of both antigens. By expressing a synNotch receptor with low-affinity for a given antigen, which controls the expression of a high affinity CAR against the same antigen, Hernandez-Lopez et al. recently engineered T cells to discriminate with high sensitivity between tumor cells overexpressing HER2 and normal cells displaying low HER2 density (69).

In 2017, Nissim et al. presented a revolutionary AND gate comprising a two-module mRNA circuit, which they used for the in-vivo delivery of a multi-component immunostimulatory cassette to be exclusively expressed in cancer cells (51). The expression of the GOI is governed by the coordinated activity of two separate modules, each driven by one of two synthetic promoters, which are simultaneously active in cancer cells only. While operating here as a multi-functional immune modulator, this approach offers numerous gated applications in ACT.

Several strategies have exploited hypoxia-induced pathways for designing self-decision-making CAR-T cells. One such approach utilizes CARs that incorporate an oxygen-sensing subdomain (HIF-1α) into the CAR scaffold and become active only under hypoxic conditions (70). Another strategy restricts CAR expression to the TME by introducing HRE regions into the CAR promoter, thus coupling transcription to hypoxia (71).

A synthetic promoter [dubbed CARTIV (54)], places a CAR gene under the control of inflammation and hypoxia-induced signals characterizing the TME. To obtain a proof of concept, the authors incorporated into these promoters DNA elements responsive to IFNγ, TNFα and hypoxia and showed an additive effect on CAR expression and function in primary human T cells in the presence of the three stimuli.

A unique AND gate integrating inputs from the TEC-associated promoter of the pre-proendothelin-1 gene and the local presence of TNFα, directs the in-vivo expression of a suicide Fas-TNFR1 receptor selectively to TECs, exerts an antiangiogenic effect and shows promising efficacy in clinical studies (72, 73).

‘Probodies’ are soluble antitumor mAb-based prodrugs, which function as two-module AND gates (74). While the antigenic cue is targeted by the probody antigen-binding site, it is masked by a cleavable peptide, designed for preferential removal at the protease-rich TME. The therapeutic potential of probodies was initially demonstrated in a human xenograft model for non-small cell lung cancer, using the anti-EGFR antibody cetuximab (75) and was later applied to antitumor CARs, using essentially the same molecular and experimental design (76).

### NOT Logic Gates

NOT gates are designed to protect normal cells that express a selected tumor antigen targeted by an activating CAR (aCAR) or TCR), as well as a second antigen (the ‘protective’ antigen) that is NOT expressed by the tumor. An effective NOT device would counteract T cell activation triggered by the tumor antigen when simultaneously engaging the protective antigen on the same normal cell. Creating a safe NOT gate entails the incorporation of an inhibitory module specific to the second antigen, which can operate through either strong inhibitory signaling or potent competition with the activating module while guaranteeing continuous dominance over the latter. It is mandatory that the effect of the NOT module on the therapeutic T cell is transient and fully reversible, as repeated inhibitory signaling can anergize these cells and abolish their therapeutic efficacy.

The implementation of the inhibitory approach for producing a NOT gate was pioneered by Fedorov at al (77)., who recruited the signaling domains of the T cell inhibitory receptors CTLA-4 and PD-1 to create inhibitory CARs (iCARs) (**Figure 1**). Since then a number of studies exploring NOT gates have examined iCARs incorporating inhibitory signaling elements that are derived from T or NK cell-associated inhibitory receptors, including PD-1 (40, 78), BTLA (79) and LIR1 (39).

Employing competitive inhibition, the SUPRA CAR technology offers a ‘Cell Selector’ application (**Figure 1**) (59). Here, the presence of a protective antigen on normal cells recruits a designated zipFv harboring a zipper, which binds avidly to the activating zipFv zipper, blocking its binding to the zipCAR, thereby preventing activation. Another competition-based NOT module is offered by the Co-LOCKR system (**Figure 1**), allowing the simultaneous recognition of antigen A AND antigen B but NOT antigen C, creating an AND-NOT gate (62). This scenario is addressed through a third soluble component specific to antigen C, which carries a ‘Decoy’ segment. Upon antigen binding on the cell to be protected, the Decoy binds tightly to the adjacent ‘Key’ and prevents binding of the latter to the ‘Cage’, so that release of the recruitment peptide and subsequent T cell activation cannot take place. Another antigen-driven AND-NOT circuit was recently reported (**Figure 1**), utilizing a synNotch receptor, which governs the expression of the pro-apoptotic factor tBID (truncated BID, a member of the BH3-domain-only subgroup of BCL-2 family) upon antigen binding as a novel, gated suicide mechanism (66).

## Conclusions

Exploiting combinations of shared antigenic and non-antigenic features characterizing all types of cancer by logic-gated technologies can dramatically increase the fidelity of tumor targeting and alleviate safety concerns. While advancing exciting gates that combine universal CARs with soluble proteins (Co-LOCKR, SUPRA CARs, RevCARs) still requires extensive calibration and optimization, others, such as split recognition or iCARs seem closer to clinical evaluation. New and powerful bioinformatic algorithms, rapidly growing datasets of tumors vs. normal cells, the development of sophisticated synthetic biology tools, along with progress made in our understanding of the intricate immune recognition and signaling networks, all pave the way for the creation of increasingly powerful logic gates for improved precision of cancer immunotherapy.

## Author Contributions

MS and GG wrote the initial text and prepared the accompanying figure. HW-M carefully reviewed the text, made constructive comments, and helped shape the final version of the manuscript. All authors contributed to the article and approved the submitted version.

## Conflict of Interest

The authors declare that the research was conducted in the absence of any commercial or financial relationships that could be construed as a potential conflict of interest.

## Publisher’s Note

All claims expressed in this article are solely those of the authors and do not necessarily represent those of their affiliated organizations, or those of the publisher, the editors and the reviewers. Any product that may be evaluated in this article, or claim that may be made by its manufacturer, is not guaranteed or endorsed by the publisher.
